# A novel syndrome of paediatric cataract, dysmorphism, ectodermal features, and developmental delay in Australian Aboriginal family maps to 1p35.3-p36.32

**DOI:** 10.1186/1471-2350-11-165

**Published:** 2010-11-19

**Authors:** Kathryn Hattersley, Kate J Laurie, Jan E Liebelt, Jozef Gecz, Shane R Durkin, Jamie E Craig, Kathryn P Burdon

**Affiliations:** 1 Department of Ophthalmology, Flinders University, Adelaide, SA, 5042, Australia; 2 Department of Genetics and Molecular Pathology, SA Pathology, South Australia, Australia; 3 Schools of Paediatric and Reproductive Health and Molecular and Biomedical Sciences, University of Adelaide, Adelaide, Australia

## Abstract

**Background:**

A novel phenotype consisting of cataract, mental retardation, erythematous skin rash and facial dysmorphism was recently described in an extended pedigree of Australian Aboriginal descent. Large scale chromosomal re-arrangements had previously been ruled out. We have conducted a genome-wide scan to map the linkage region in this family.

**Methods:**

Genome-wide linkage analysis using Single Nucleotide Polymorphism (SNP) markers on the Affymetrix 10K SNP array was conducted and analysed using MERLIN. Three positional candidate genes (*ZBTB17, EPHA2 *and *EPHB2*) were sequenced to screen for segregating mutations.

**Results:**

Under a fully penetrant, dominant model, the locus for this unique phenotype was mapped to chromosome 1p35.3-p36.32 with a maximum LOD score of 2.41. The critical region spans 48.7 cM between markers rs966321 and rs1441834 and encompasses 527 transcripts from 364 annotated genes. No coding mutations were identified in three positional candidate genes *EPHA2, EPHB2 *or *ZBTB17*. The region overlaps with a previously reported region for Volkmann cataract and the phenotype has similarity to that reported for 1p36 monosomy.

**Conclusions:**

The gene for this syndrome is located in a 25.6 Mb region on 1p35.3-p36.32. The known cataract gene in this region (*EPHA2*) does not harbour mutations in this family, suggesting that at least one additional gene for cataract is present in this region.

## Background

The identification of genes for syndromic disorders not only provides insights into the cause and molecular pathology of disease but also advances our understanding of their normal function. Although technology has advanced to the point where large-scale genome-wide association studies for complex diseases are now feasible, family based disease gene discovery still plays an important role, particularly in identifying the genetic causes of Mendelian disease [1]. Even in the case of rare syndromes, patients and families benefit from a clear molecular diagnosis which may assist with prognosis and clinical management as well as risk prediction enabling prenatal and preimplantation genetic diagnosis [2]. In addition, novel disease gene discovery can lead to insights into the function of those genes and a better understanding of the underlying biology [3,4]. Many syndromes have been described where the causative gene mutation has yet to be identified.

We recently described a novel phenotype in a large family of Australian Aboriginal descent [5]. The phenotype is characterised by developmental delay, short stature, childhood onset cortical cataract, facial dysmorphism, clinodactyly, thin hair and an erythematous skin rash. The syndrome was identified in 5 male children, related at the level of second cousins. The two mothers of the 5 affected male children displayed only a mild cortical cataract not yet requiring surgery and are of short stature, but do not display the other features. The cataract in affected males was severe requiring surgery during early childhood. Initial inspection of the pedigree suggested X-linked inheritance. Linkage analysis of the X chromosome failed to detect any region of the chromosome identical-by-descent in the 5 children, excluding linkage from this chromosome [5] and therefore suggesting autosomal dominant inheritance. Thus we have conducted a genome-wide linkage analysis to localise the gene for this distinctive phenotype.

## Methods

This project was approved by the Flinders Clinical Research Ethics Committee and the Research Ethics Committee of the Women's and Children's Hospital, Adelaide, South Australia. All participants and/or their guardian gave informed consent. As described previously [5], DNA was extracted from peripheral whole blood of all available family members. Simulations for maximum LOD score were conducted in SLINK [6]. All clearly diagnosed cases and their parents and siblings (when available) were genotyped on the Affymetrix 10K SNP array at the Australian Genome Research Facility using standard protocols. This array contains ~10,000 Single Nucleotide Polymorphisms (SNPs). Due to uncertain affection status in individuals III:5, III:6 and IV:6, they were not included in the initial genome-wide scan. Linkage analysis was conducted using the MERLIN program [7]. Any errors in the genotyping were detected using the error checking mode of MERLIN and removed using PEDWIPE before a multipoint linkage analysis was carried out. A fully penetrant autosomal dominant model was used with a rare disease frequency of 0.0001. Haplotypes were also reconstructed in MERLIN.

Six polymorphic SNPs that defined the haplotypes in the linked region were selected (rs580309, rs718206, rs720035, rs1416464, rs216036 and rs372151) and genotyped using SNaPshot^® ^in all available family members to determine the carrier status of the linked region in untyped individuals. Previously typed individuals were also re-genotyped to ensure correlation between the two genotyping methods. Briefly, a primer directly adjacent to the polymorphic nucleotide was annealed to the PCR product, and a single fluorescently tagged ddNTP was incorporated according to the manufacturer's protocols (Applied Biosystems). The products were then electrophoresed on the ABI PRISM 3100 Genetic Analyzer to detect the alleles.

To identify genes from the linkage interval which are likely to be expressed in the eye, we searched the NEIbank[8] database. Genes were classified as either not expressed in eye or expressed in one of three categories; "eye" (includes libraries from whole eye, retina, cornea etc), "fetal eye" and "lens". The categories were not exclusive. All genes expressed in "lens" are also in "eye".

Candidate genes were chosen from the linked region on the basis of putative function, expression pattern or association with congenital cataract. Primers were designed to amplify the coding exons (including splice sites) of each gene, and PCR products were directly sequenced on the ABI PRISM 3100 Genetic Analyzer (Applied Biosystems, Foster City, CA) with BigDye Terminators (Applied Biosystems) according to standard protocols. Each exon was sequenced in two affected individuals, and sequences were compared to the reference sequence.

## Results

**Figure 1:**
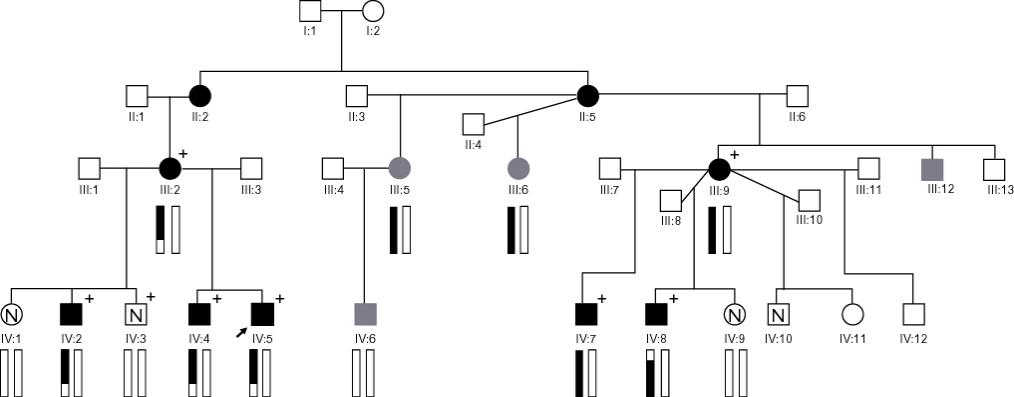
**Pedigree drawing showing haplotypes on chromosome 1p for genotyped family members**. The segregating haplotype is coloured black. All other haplotypes are white. Examined unaffected individuals are marked "N". Grey symbols indicate "unexamined but reportedly affected" individuals. The proband IV:5 is marked by an arrow. A "+" symbol indicates those included in the genome-wide scan. Recombinant individuals III:2 and IV:8 define the critical region between markers rs966321 and rs1441834.

Eight family members including 5 boys with a clear diagnosis were available for the initial genome-wide analysis (Figure 1). Affected status was determined as bilateral cataract as a minimal requirement, but all 5 affected boys also displayed dysmorphism, erythematous rash and developmental delay as described previously [5]. Unexamined individuals III:5, III:6 and IV:6 were not included in the analysis as they were not available for detailed clinical evaluation, making a definitive diagnosis in these individuals difficult. Individual III:5 was reported to have had a unilateral cataract removed duing early adulthood, but no further information was available. Similarly, some developmental delay was reported in her son IV:6, but this was unable to be verified, and he was not available to have a formal cataract examination.

**Figure 2:**
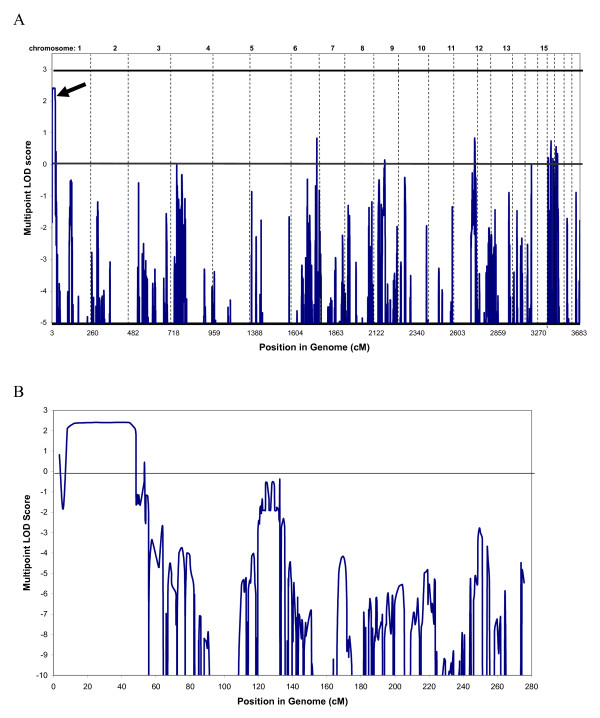
**Linkage analysis**. A) Multipoint genome-wide LOD scores. Linkage was calculated in MERLIN using family members marked with "+" on Figure 1, under a fully penetrant dominant model. Horizontal line marks LOD = 3.0. Arrow indicates linkage peak on chromosome 1p. B) Multipoint LOD scores on chromosome 1. The peak extends from SNP rs966321 to rs1441834 (8.0-56.7 cM), Max LOD = 2.41.

Simulation studies indicated that this family would generate a maximum LOD score of ~2.4 using the samples available. Multipoint genome-wide linkage analysis identified linkage to the telomere of chromosome 1 with a maximum multipoint LOD score of 2.41 (Figure 2) using the family members with a clear diagnosis and for whom sufficient DNA was available. No other regions of suggestive linkage were detected. Haplotype estimation identified recombination events in individuals III:2 and IV:8 which define the critical region to a 48.7 cM region between rs966321 and rs1441834 on 1p35.3-p36.32 (Figure 1). The defining markers are ~50 kb and ~820 kb respectively from the next typed marker, thus suggesting that additional fine mapping, particularly at the proximal end could be of value. However, at the distal end there is only one annotated gene (*AJAP1*) between rs966321 and the next linked marker SNP rs1599169. Similarly, at the proximal end there are no annotated genes between linked marker rs446335 and defining marker SNP rs1441834. Thus, additional fine mapping could only hope to exclude this one gene from the critical region and thus was not considered a high priority.

**Figure 3:**
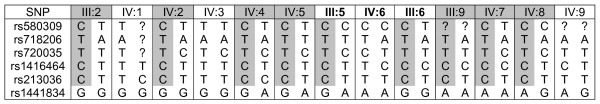
**Phased haplotypes for all genotyped members of the family**. Affected individuals and the segregating haplotype are shaded grey. Participants of uncertain diagnosis and carrier status are marked in bold. Both III:5 and III:6 clearly carry the segregating disease haplotype while IV:6 likely does not. Unaffected individuals IV:1, IV:3 and IV:9 do not carry this haplotype. "?" indicates allele not scored.

In order to determine if individuals III:5, III:6 and IV:6 also carry the disease linked chromosome, a selection of six polymorphic markers from across the linkage region were typed in the whole family. The haplotypes for the six markers were constructed (Figure 3), and show that while III:5 and III:6 are carriers of this disease chromosome, IV:6 is not, having inherited the grandpaternal chromosome from his mother.

The 25.6 Mb linked region contains 531 transcripts from 364 RefSeq annotated genes extracted from the USCS Genome Browser, HG19. As cataract has been used as a characteristic feature on which to base the designation of "affected", the expression pattern of each gene in lens and fetal eye was investigated *in silico *through the resources of NEIbank. Of these genes, 136 are not reported to be expressed in eye tissue. Another 140 were found in eye tissues in general, 166 were reported in fetal eye libraries and 85 in the lens.

While selection of candidate genes from such a long list of positional candidates is difficult, we noted that one candidate gene in particular, *ZBTB17 *is expressed in skin as well as lens. Given the rash observed as part of this syndrome this gene became an attractive candidate. ZBTB17 (zinc-finger and BTB domain containing protein 17, myc-interacting protein 1) is a transcription factor that associates with the microtubules of the cytoskeleton [9], and interacts with the proto-oncogene *myc *[10]. ZBTB17 activates gene transcription in response to changes in the cytoskeleton and in response to cell-cell adhesion formation [9,11]. Correct cell adhesion and morphology is vital to maintaining the transparency of the ocular lens. ZBTB17 and myc interact to control the transcription of many genes [10]. They are important in maintaining homeostasis of skin cells and keratinocytes, inducing skin stem cells to exit from the cell cycle and differentiate [12]. All coding regions of the *ZBTB17 *gene were sequenced in two affected family members but no mutations were identified.

Recently, we and others reported that mutations in the *EPHA2 *gene cause both congenital [13,14] and age-related cataract [15]. The tendency of EPHA2 to be associated with the cortical subtype of cataract in adults was of particular interest as the affected members of this family displayed the unusual phenotype of juvenile onset progressive cortical cataract. EPHA2 is a receptor for ephrin-A5 (EFNA5). Mice deficient in EFNA5 also develop cataract [16]. The eph-ephrin signalling pathways are involved in numerous cellular processes including cell morphogenesis, tissue patterning, cell migration, cell adhesion, cell proliferation and cell-fate determination. There are a variety of different EPH receptors and ephrin ligands. Also found within the linked region in this family is the *EPHB2 *gene, which along with the known cataract gene *EPHA2 *is a receptor for EFNA5. Thus, we directly sequenced all exons and intron-exon boundaries of both *EPHA2 *and *EPHB2 *in this family. No mutations were identified in either gene, indicating that coding mutations are not responsible for the phenotype in this family.

## Discussion

This study has identified a region on chromosome 1p36 linked to a distinctive phenotype consisting of cortical cataract, developmental delay, short stature, facial dysmorphism, clinodactyly, thin hair and an erythematous skin rash. Many other syndromes and diseases have also been shown to link to this region, some of which show significant phenotypic overlap with this family. Notably, terminal or interstitial deletions of 1p36 show a spectrum of features with marked similarity [17]. Nearly 100% of such patients display mental retardation and developmental delay. Characteristic dysmorphic features observed in the majority of this group of patients include large anterior fontanelle, microcephaly, brachycephaly, dysmorphic ears, flat nasal bridge, pointed chin and clinodactyly. Conductive hearing loss is also reported and is present in the family reported here. It is highly probable that many of these overlapping features are contributed to by the deletion of the gene responsible for the phenotype in this family. Cataract is not commonly reported in 1p36 deletion patients, although other eye anomalies such as hypermetropia, myopia and strabismus are relatively common. This may be due to the effect of monosomy compared with a mutation that disrupts normal protein function.

It is highly unlikely that this family carries a contiguous gene deletion on 1p36 by virtue of the heterozygosity observed throughout the linked region in all affected individuals, although microdeletions in between markers cannot be ruled out. Array based comparative genomic hybridisation (aCGH) previously failed to identify any copy number variants with a resolution of 0.2-1.0 Mb [5]. Ten of the 63 linked markers show homozygosity in all affected individuals but only two of these markers are contiguous (rs2319403 and rs2319404). These two SNPs are located only 131 bp apart in an intron of the *PTPRU *gene. This gene is a plausible candidate as it is reported to be expressed in the crystalline lens and is involved in cell-cell adhesions, interacting with beta-catenin and E cadherin [18]. The next flanking heterozygous markers define a region of nearly 380 kb containing five genes (*EPB41, TMEM200B, SFRS4, MECR *and *PTPRU*) all of which are expressed in lens. A microdeletion in this region encompassing the two homozygous markers and thus disrupting at least the *PTPRU *gene cannot be completely ruled out with this methodology.

**Figure 4:**
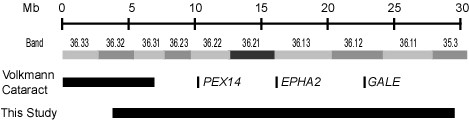
**Schematic of the linkage region**. Overlap of the linkage region in this family with the mapped Volkmann Cataract region is shown along with the location of other genes causing cataract phenotypes (*PEX14, EPHA2 *and *GALE*)

Other known syndromes mapping to 1p36 with phenotypic overlap with this family include Zellweger syndrome, galactose epimerase deficiency, and congenital and age-related cataract. Zellweger Syndrome (OMIM #214100) is a fatal recessive disease affecting the brain, liver and kidneys [19]. It is caused by disruption to peroxisome biogenesis. Many genes are known to cause the disease, including *PEX14 *on 1p36 [20]. This gene is known to be expressed in the eye and some reports of Zellwegger syndrome describe ocular features. Notably, parents of affected babies (i.e. heterozygous carriers of the condition) have been shown to have mild cataract, described as "curvilinear cortical condensations" [19], although comparison of the photographs published by Hittner *et al *[19] do not resemble the cataract observed in this family [5]. Galactose epimerase deficiency (OMIM #230350) is another syndrome mapping to 1p36 that can lead to cataracts. Deficiency of the UDP-galactose-4-epimerase (*GALE*) gene results in a form of galactosemia, with an inability to metabolise galactose and increases the risk of cataracts. Some patients also display developmental delay. Both age-related cortical cataract (OMIM #613020) and congenital posterior polar or total cataract (OMIM #116600) have been linked to 1p36 with the *EPHA2 *gene recently being described as causative of both conditions [13-15]. Coding mutations of *EPHA2 *have been ruled out in the family in the current report, but intronic or distant regulatory mutations remain a possibility. A second locus for congenital cataract (Volkmann type, OMIM #115665) has also been reported on 1p36 [21]. The linkage region does not encompass the *EPHA2 *gene, however it does overlap with the region described in this Australian family (Figure 4). Although the Volkmann cataract phenotype is not syndromic and is likely distinct from that described in this family, it is possible that the two are allelic.

The number of potential candidate genes for this phenotype in the linked region is very large. While expression information is useful in prioritising genes for analysis, the number of genes to assess in this particular case remains large, and expression profiles generated by EST libraries and micorarray information are not exhaustive, meaning even more genes are potential candidates. It was initially thought that this pedigree displayed X-linked inheritance, due to a milder phenotype observed in females III:2 and III:9. However, a thorough evaluation of the X chromosome ruled out this possibility [5], which was confirmed by re-analysis of the X chromosome in this genome-wide study. The reason for the differential expression in the females of generation III and the males of generation IV is not clear. Anticipation may be an explanation for generational differences. It is also possible that the gene responsible is regulated differently in males and females or that the gene product interacts with other factors (eg sex hormones) present at different levels in males and females. Alternatively, the genetic background may be important for phenotype expression, with cousins III:2 and III:9 carrying a common inherited protective allele at an independent interacting locus not passed on to the affected offspring.

## Conclusion

We have localised the region for a novel phenotype characterised by cataract, mental retardation, erythematous rash and facial dysmorphism to a region on chromosome 1p36. Further evaluation of all linked genes is required to identify the causative gene.

## Competing interests

The authors declare that they have no competing interests.

## Authors' contributions

KH conducted the linkage analysis and genotyping of SNPs in the linked region, conducted the bioinformatic analysis of linked genes and contributed to preparation of the manuscript. KJL sequenced the candidate genes. JEL, JG, SRD and JEC were involved in the clinical diagnosis and recruitment of family members. JG and JEC were also involved in manuscript preparation. KPB designed the study, oversaw laboratory experiments and analysis and drafted the manuscript. All authors read and approved the final manuscript.

## Pre-publication history

The pre-publication history for this paper can be accessed here:

http://www.biomedcentral.com/1471-2350/11/165/prepub
